# A Cost-Utility Analysis of Mesh Prophylaxis in the Prevention of Incisional Hernias following Stoma Closure Surgery

**DOI:** 10.3390/ijerph192013553

**Published:** 2022-10-19

**Authors:** Yusuf Sheikh, Hareef Asunramu, Heather Low, Dev Gakhar, Keerthi Muthukumar, Husam Yassin, Laure de Preux

**Affiliations:** 1 Faculty of Life Sciences and Medicine, King’s College London, London WC2R 2LS, UK; 2 Faculty of Medicine, Imperial College London, London SW7 2DD, UK; 3 Faculty of Medical Sciences, University College London, London WC1E 6DE, UK; 4 Department of Economics and Public Policy, Business School, Imperial College London, London SW7 2AZ, UK

**Keywords:** stoma closure, incisional hernia, mesh prophylaxis, cost-utility analysis

## Abstract

Background: Stoma closure is a widely performed surgical procedure, with 6295 undertaken in England in 2018 alone. This procedure is associated with significant complications; incisional hernias are the most severe, occurring in 30% of patients. Complications place considerable financial burden on the NHS; hernia costs are estimated at GBP 114 million annually. As recent evidence (ROCSS, 2020) found that prophylactic meshes significantly reduce rates of incisional hernias following stoma closure surgery, an evaluation of this intervention vs. standard procedure is essential. Methods: A cost-utility analysis (CUA) was conducted using data from the ROCSS prospective multi-centre trial, which followed 790 patients, randomly assigned to mesh closure (*n* = 394) and standard closure (*n* = 396). Quality of life was assessed using mean EQ-5D-3L scores from the trial, and costs in GBP using UK-based sources over a 2-year time horizon. Results: The CUA yielded an incremental cost-effectiveness ratio (ICER) of GBP 128,356.25 per QALY. Additionally, three univariate sensitivity analyses were performed to test the robustness of the model. Conclusion: The results demonstrate an increased benefit with mesh prophylaxis, but at an increased cost. Although the intervention is cost-ineffective and greater than the ICER threshold of GBP 30,000/QALY (NICE), further investigation into mesh prophylaxis for at risk population groups is needed.

## 1. Introduction

### 1.1. Background

Stomas are openings made during surgery that connect the bowel to the abdominal wall to allow waste to be diverted out of the body and are common in patients suffering from irritable bowel disease, diverticulitis and colorectal cancer [1]. The majority of stomas performed are ileostomies or colostomies [2]. Stoma formation can be either temporary or permanent, with temporary ‘loop stomas’ requiring surgical reversal usually 2 to 3 months later [3]. Stoma closure is necessary for patients wishing to regain normal bowel function and a reduction in stoma associated morbidity [4]. Surgical closure of the stoma site with sutures is considered gold-standard, yet complications such as wound infection and seroma formation are frequent. Many of these complications, like wound infection, are key risk factors for wound breakdown, which directly contribute to the development of incisional hernias. The end result of incisional hernias is an accumulating incidence of pain, reoperation, and emergency surgery due to complications such as bowel strangulation [5]. Biological mesh prophylaxis has been suggested to reduce hernia incidence following stoma closure, yet the current evidence base is limited. The ROCSS trial provided the first high-quality evidence for the benefit of providing mesh prophylaxis during stoma closure [6].

### 1.2. Motivation and Rationale

Stoma closure is a widely performed procedure, with 6295 undertaken in England in 2017 alone [7]. However, stoma closure is associated with significant complications (with a complication rate ranging between 20–70%), of which incisional hernia is the most severe, occurring in 30% of patients [2,8]. Treatment of complications place considerable financial burden on the NHS, with hernia costs to the NHS estimated at £114 million annually, although this is likely to be a conservative estimate [9]. As recent evidence has found that using a prophylactic mesh significantly reduces the rate of incisional hernias following stoma closure, an economic evaluation of the costs and benefits of this intervention compared to standard procedure is essential [6].

### 1.3. Study Objectives

This study aims to conduct a cost-utility analysis (CUA), comparing stoma closure with mesh prophylaxis to stoma closure with sutures alone, for prevention of incisional hernias post-surgery. By using UK monetary and health benefit data, this study will provide recommendations to inform NICE guidelines that maximise the efficiency of NHS resources.

### 1.4. Literature Review

A systematic literature search was conducted on the 9th of February 2021 using the electronic databases EMBASE and MEDLINE (Appendix A). The following keywords “prophylactic mesh”, “stoma”, “hernia”, and relevant synonyms were used to formulate a search string. Exclusion and inclusion criteria are also reported (Appendix B*)*. The search revealed that no economic evaluation has been performed for this intervention in stoma closure, making this a novel analysis. Subsequent grey literature searching revealed a cost-effective analysis using Canadian data, which found mesh prophylaxis was dominant compared with no mesh for a different procedure (colostomy formation), using Canadian data [10]. However, the results of this economic analysis are not generalisable for patients undergoing stoma closure. 

A recent systematic review and meta-analysis identified three studies investigating the efficacy of mesh prophylaxis in stoma closure for prevention of incisional hernias [11]. Of the included studies, two were retrospective cohort studies [12,13] and one was a prospective study [14]. Although all three studies reported a significantly lower risk of incisional hernia with prophylactic mesh usage compared to without (in line with the findings from ROCSS), these findings must be considered in the context of key study limitations. 

Both Liu and Warren’s studies are inherently limited by their retrospective designs. In Liu’s study, variation in stoma closure techniques between the intervention and control groups may have confounded results. In Warren et al’s study, the large difference in sample size between the mesh and control arms (91 and 268 patients, respectively) questions both the accuracy and generalisability of the findings reported. A key limitation in Maggiori’s study was the heterogeneity of its population (which included cancers, IBD and bowel obstructions), resulting in varying risks of postoperative hernia between the two groups. Furthermore, clinical outcomes reported were from Australian, French, and American patient populations, respectively, reducing generalisability to the UK population. Finally, hernia incidence following stoma closure increases over time–the aforementioned studies all had short follow-up times (average 14 months); thus, it is possible that hernia incidence was underestimated. 

Bhangu et al.’s randomised controlled trial (ROCSS) published in the Lancet marks the most recent and comprehensive investigation into this area. This trial was performed across 37 European hospitals (35 UK hospitals) and compared the clinical outcomes of patients undergoing stoma closure with a prophylactic mesh to a control group (closure without a mesh). The trial consisted of 790 patients, randomly assigned to mesh closure (394 patients) and standard closure (396 patients), The primary outcome measured was the occurrence of clinically detectable hernia at 2 years post-randomisation–the longer time period analysed makes the findings from the ROCSS trial considerably more reliable than those in previous studies. The trial reported significantly lower rates of incisional hernia in the mesh group (12%) compared to the no mesh group (20%), providing the first high-quality evidence for benefit in providing mesh prophylaxis. However, a formal cost evaluation is required to inform NICE guidelines for prevention of incisional hernia following stoma closure. Cost and benefit data for this economic analysis is acquired from the ROCSS trial [6].

## 2. Materials and Methods

### 2.1. Choice of Analysis

In this economic evaluation, a cost-utility analysis was undertaken with cost measured in monetary units (GBP) and utility in Quality Adjusted Life Years (QALYs), using mean EQ-5D-3L scores from the ROCSS trial. The QALY is the most commonly used measure of health in a CUA, combining the attributes of length and quality of life into a standardised measure, enabling comparison of healthcare interventions for optimal resource allocation [15,16]. Moreover, NICE uses QALYs to determine healthcare resource allocation, thus QALYs are the most suitable unit for a CUA based on the NHS perspective [17]. This is considered preferable to a cost-effective analysis (CEA), which is limited to the comparison of physical units, e.g., length of life gained. A cost–benefit analysis (CBA) was not performed due to the uncertainty in the monetary valuation of health benefits.

### 2.2. Choice of Perspective

This economic evaluation has thus been conducted from the perspective of the NHS, solely considering the costs incurred by the NHS. In light of the COVID-19 pandemic, the strain on the UK’s National Health Service (NHS) and the need for optimal resource allocation has never been greater [18]. Stoma closure is a common elective procedure associated with frequent complications. Cost of treatment impacts both primary and secondary care services, hence the rationale for assessing the cost-utility of this intervention from the NHS perspective is justified.

### 2.3. Competing Alternatives

The intervention (prophylactic biological mesh: non-crosslinked porcine collagen tissue matrix) was compared to standard closure (sutures alone) of stoma site. Current international guidelines do not recommend routine use of prophylactic biological mesh for prevention of incisional hernias during stoma site closure [6]. However, with 30% of patients suffering hernias following stoma closure, the effectiveness of current techniques is highly questionable [8]. Previous trials have investigated the use of cheaper synthetic meshes for the prevention of incisional hernias; however, this alternative is associated with higher rates of wound infection and other complications than biological meshes [14]. Furthermore, although various surgical techniques have been explored to reduce complications during stoma closure, current evidence on their efficacy is both limited and dependent on surgical training [19]. As such, the use of a biological prophylactic mesh was deemed the most suitable comparator to standard closure for this investigation.

### 2.4. Time Period

The ROCSS trial’s primary outcome (incidence of clinical hernias) was measured at 2 years (24 months) post stoma closure, in both the mesh and non-mesh groups. Though the majority of hernias form within 2 years of surgery, this timepoint is considered too early for the full spectrum of complications to occur [20]. A 2-year analytic horizon was thus used for this evaluation as further complication data was not available beyond this time frame.

### 2.5. Costs

#### 2.5.1. Procedure and Intervention Costs

Current NHS practice does not utilise mesh prophylaxis. A procedural cost (GBP 4247.60) for stoma closure with sutures was obtained from the NHS reference costs. The intervention used in the ROCSS trial was a biological prophylactic mesh inserted during closure of the stoma site. The cost of biological meshes vary depending on weight, size, and manufacturer. An average mesh cost of GBP 1650 was obtained from a cost-effectiveness analysis of biological meshes for an alternative procedure (stoma formation surgery) using UK manufacturer data [21]. Additional costs as a result of the increased operating time associated with mesh insertion were also considered, providing a total intervention cost of GBP 6597.01 (See Appendix C for complete cost breakdown). 

#### 2.5.2. Complications Costs

In the ROCSS trial, the primary outcome reported was the incidence of clinical hernia. Secondary outcomes included seroma formation and wound infection. In this economic evaluation, a full breakdown of the costs involved in the treatment and management of a hernia, seroma, and wound infection was required. Analysis of existing literature revealed that 51% of hernias and 6.7% of seromas would require treatment [22], while all wound infections would require dressing, antibiotics, and a GP visit. The rate of treatment for each complication was then multiplied by the cost of the associated procedures (Appendix D).

#### 2.5.3. Assumptions

In calculating the cost of specific complications, the following assumptions were made:

- A cost for seroma drainage was not obtainable; instead the procedural cost for an abscess drainage was sourced from the NHS reference costs and utilised as a proxy. This is justified as the management of a seroma and abscess are the same, with both requiring single, percutaneous abdominal drainage.
- NHS reference costs for stoma closure, abscess drainage and hernia repair surgery are dependent upon patient comorbidity and complication (CC) scores. Based on the patient data in the ROCSS study, a CC score of 2 was assigned to all patients and the corresponding procedural cost was used.
- Diagnostic costs for each complication were omitted as these could not be reliably estimated based on the study data. This is justified as the majority of complications in this study can be visibly diagnosed.

#### 2.5.4. Viewpoints and Net Present Values

For all complications assessed, costs to the NHS were considered from primary and secondary care viewpoints, including both hospital costs and GP outpatient visits. All costs were sourced from UK-based market data, the NHS reference costs or the British National Formulary (BNF) [23]. Historical costs were compounded at a rate of 3.5% in accordance with current NICE guidelines to provide present values for 2021.

### 2.6. Benefits

Benefit data was obtained from the results of the ROCSS trial. Mean EuroQol EQ-5D-3L scores were reported at 30 days post-operation, and 1- and 2-years post-randomisation. The EuroQol EQ-5D-3L score ranges from 0 to 1, with a higher score indicating greater quality of life. This outcome measure was selected as it can be used to calculate QALYs, enabling comparison to other treatments provided by the NHS. The ROCSS trial reported a mean EQ-5D-3L score of 0.79 and 0.81 at 30 days, 0.86 and 0.84 at 1 year, 0.85 and 0.85 at 2 years post-randomisation for the mesh and no mesh groups, respectively. An overall QALY for the 2-year period was then calculated using the mean EQ-5D-3L scores reported at each time period (see Appendix E for the full QALY calculations). Importantly, this mean included patients experiencing both complications and hernias. Both seroma formation and wound infection are short-term complications, thus the assumption that they have no impact on QALYs is medically justifiable. However, the study data omits utilities for having a hernia, which may have considerable impact on patient quality of life, hence a sensitivity analysis was conducted on this later on. Discounting was not required as the value of a QALY remains stable over time.

### 2.7. Modelling

**Figure 1:**
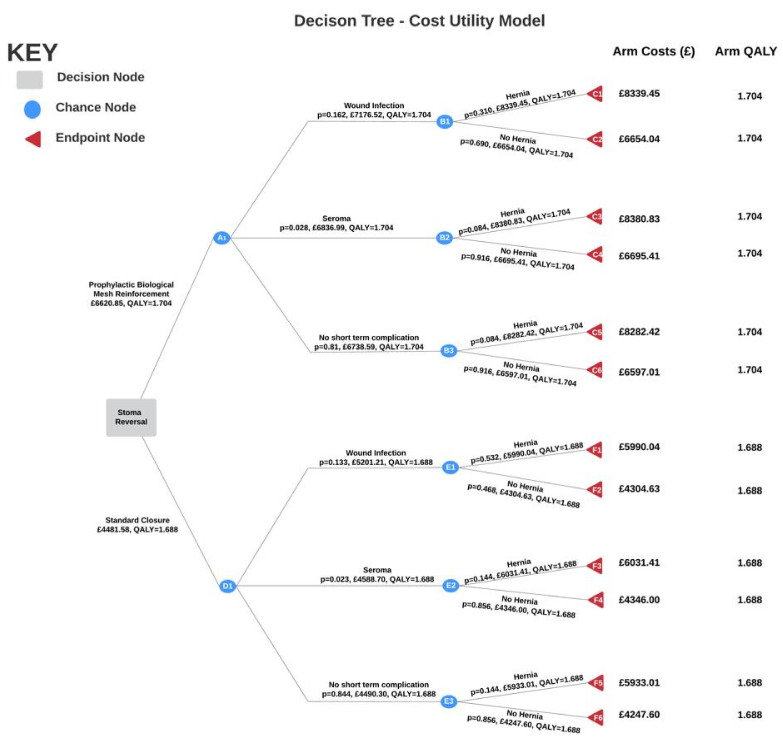
The decision tree compares prophylactic biological mesh reinforcement with standard suture closure.

The trial reported 1 primary outcome and 6 secondary outcomes. Only the clinically relevant outcomes were included in the decision tree (Figure 1), namely the primary outcome, incidence of clinically detectable hernia, and two secondary outcomes, wound infection, and seroma formation. Appendix F explains why the other secondary outcomes were not included in the decision tree model. As the ROCSS trial only reports mean quality of life and hernia incidence outcomes for the entire sample, it was not possible for sub-stratification of the study population based on primary diagnosis and thus no grouping of clinical outcomes was performed. However, given randomization, it is likely that the distribution of conditions does not vary significantly between the control and experimental groups within the study. Furthermore, as the mean age of trial patients was 58.7 years, the sample studied is representative of the normal population requiring stoma reversal.

In modelling the data, the following assumptions were made:

- The ROCSS study data suggests that the probability of developing an incisional hernia is independent of any complications, which is contradictory to hernia pathophysiology. Wound infection directly contributes to development of hernia [6]. Using our medical knowledge and existing literature, a probability of hernia given wound infection was calculated (see Appendix G).
- The probabilities for both wound infection and seroma formation are mutually exclusive. This is justified as the ROCSS trial did not provide conditional probabilities for each complication.
- EQ-5D-3L scores for wound breakdown, seroma and no complication in this study are the same for each arm. Although the mean EQ-5D-3L scores provided in the study ignore the impact of complications, however these complications are short-term and are therefore unlikely to be detrimental to quality of life.
- Although death is a consideration when conducting surgical procedures, no patient deaths were reported in the study, so deaths were not investigated as an outcome in the tree. Moreover, the NHS reports stoma reversal surgery as a relatively straightforward procedure with a low probability of serious complications [24].

To obtain an overall cost for the endpoint of each branch, the cost of the intervention was added to the cost of complications of each branch. For example, a patient in the intervention group who experienced a wound infection and then a hernia would incur a total cost of GBP 8339.45 (GBP 6597.01 + GBP 57.03 + GBP 1685.41). A similar method was utilised in the calculation of the overall benefit for the endpoint of each branch.

To derive the expected values at the decision node, endpoint costs and QALYs were multiplied by the probabilities of each branch. The decision tree shows that the expected costs for the 2-year period is GBP 6620.85 for the mesh arm and GBP 4481.58 for the standard closure arm. The expected QALY is 1.704 for the mesh arm and 1.688 for the standard closure arm.

## 3. Results

### 3.1. Incremental Cost-Effectiveness Ratio

The cost and benefit data associated with the two treatment option arms was used to calculate an incremental cost-effectiveness ratio (ICER). An ICER indicates the cost-effectiveness of an intervention [25]. The ICER was compared to NICE’s “cost-effective” ceiling of GBP 30,000/QALY.

The *ICER* is outlined in the equation below:

$$ICER=\frac{COST\left(Mesh\;Prophylaxis\right)-COST\left(Standard\;Closure\right)}{QoL\left(Mesh\;Prophylaxis\right)-QoL\left(Standard\;Closure\right)}\;=\frac{£6620.85-£4481.58}{1.7041-1.6875}=GBP\;128,356.25/QALY$$

An *ICER* of GBP 128,356.25/QALY was obtained, which can be interpreted as a cost of GBP 128,356.25, for every QALY gained. The intervention provides an increased benefit but at an increased cost. The cost of the intervention is considerably greater than the cost-effectiveness threshold of GBP 30,000/QALY, rendering the intervention cost ineffective. 

### 3.2. Net Monetary and Health Benefit

It is helpful to supplement the interpretation of the ICER in the context of the quadrant of ∆C − ∆E plane to which it corresponds using additional metrics. The two other ways are the Net Monetary Benefit (NMB) and the Net Health Benefit (NHB). 

Using the typical NICE threshold of GBP 30,000/QALY, the NMB of the suggested intervention is −GBP 1639.27 and the NHB is −0.055 QALYs. As these values are smaller than 0, the intervention is judged to be cost-ineffective, pending sensitivity analysis. The NMB value means that when the maximum cost of a QALY is set at GBP 30,000, prophylactic biological mesh reinforcement is GBP 1639.27 too expensive for the level of benefit (≈0.017 QALYs) it achieves. The value of NHB tells us that at the current threshold and cost of the intervention, the health benefit of prophylactic biological mesh reinforcement falls short of being cost effective by 0.055 QALY.

### 3.3. Sensitivity Analysis

**Figure 2:**
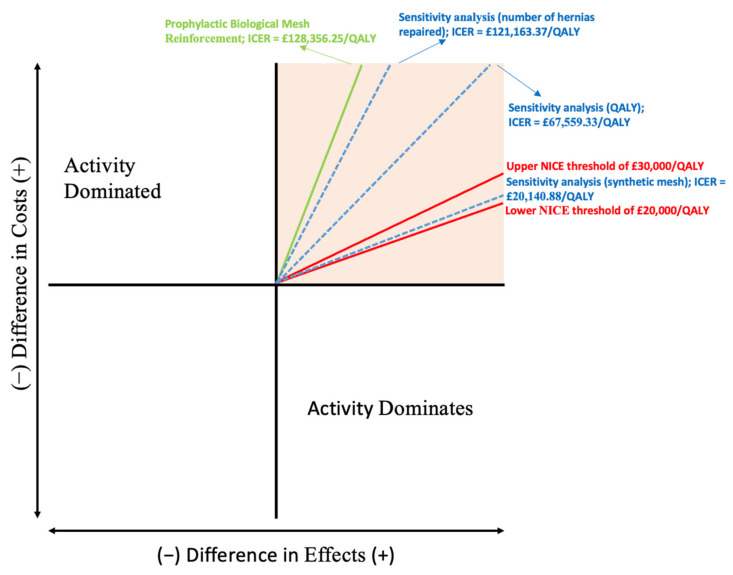
Cost-effectiveness plane for the ICER calculated between mesh prophylaxis and standard suture closure, including results from univariate sensitivity analyses.

A univariate sensitivity analysis was performed in order to test the robustness of our model, given the assumptions made (Figure 2). One at a time, parameters were changed to assess the effect it had on the value of the ICER. Preliminary analysis demonstrated that small changes in the incremental effectiveness had significant impact on the ICER. The ROCSS trial only provided the mean EQ-5D-3L scores for the two groups (mesh and no mesh). Sub-group data was not available so intra group differences in quality of life (for example between those with and without a hernia) could not be evaluated. Though hernia incidence was significantly higher in the non-mesh group, the trial showed no difference in EQ-5D-3L scores between the mesh and non-mesh groups at 2 years. However, a hernia is likely to result in a lower EQ-5D-3L score. A sensitivity analysis was carried out using utilities expected in those with and without a hernia (0.67 vs. 0.87), based on medical literature (see Appendix H). This analysis yielded a new ICER of GBP 67,559.33/QALY, indicating a cost of GBP 67,559.33 for an extra QALY gained.

Guidelines for incisional hernia repair vary globally. Currently in the NHS, incisional hernias are only indicated for treatment if there is pain/discomfort causing significant functional impairment, hence the assumption that only 51% of hernias will undergo repair. However, morbidity is likely to increase as time passes irrespective of present symptoms due to the hernia growing in size [26]. Treating all instances of hernia, a practice common in other countries, is justified by the subsequent reduction in rates of severe complications [27]. Therefore, a second sensitivity analysis was conducted based on the assumption that 100% of clinically detectable hernias will be repaired. This analysis yielded a new ICER of GBP 121,163.37/QALY, indicating a cost of GBP 121,163.37 for an extra QALY gained.

Colorectal surgeons often encounter clinical situations whereby auxiliary materials are needed to reinforce the abdominal wall. As a result, many such products have been developed, each with their own cost and health benefits, and challenges [28]. An example is evident in the case of biological and synthetic meshes. Theoretically, biological meshes are less likely to become infected and generate less of a foreign body response, however they are considerably more expensive [29]. Currently there is a lack of high-quality evidence on biological meshes, so it is difficult to discern when their use is justified [30]. Therefore, a third sensitivity analysis was conducted based on the assumption that the hernias will be repaired using the less costly synthetic mesh. This analysis yielded a new ICER of GBP 20,140.88/QALY, indicating a cost of GBP 20,140.88 for an extra QALY gained; synthetic mesh prophylaxis may provide for a cost-effective intervention, although the result should be treated with caution as it was a pure accounting exercise in this case. 

NMB and NHB calculations for these analyses can be found in Appendix I Table A9 and Table A10. Under both sensitivity analyses, the proposed intervention became more cost effective than in the original model yet remained well above the NICE threshold of GBP 30,000/QALY. The conclusions drawn from our initial cost-utility analysis are therefore robust to uncertainty.

Overall, the analysis showed that the intervention became more cost effective when the utility of patients with hernia was reduced and became more cost effective when all incisional hernias were repaired.

## 4. Discussion

The ROCSS trial found that biological mesh prophylaxis reduced hernia incidence following stoma closure, supporting findings from previous retrospective trials [12,13]. However, this cost-utility analysis found biological mesh prophylaxis to be significantly more costly than sutures alone. Although this study is the first comprehensive economic evaluation for this intervention, the findings reported align with studies evaluating the cost-effectiveness of mesh prophylaxis for alternative procedures [28]. To date, the high up-front costs for biological meshes do not justify their routine use in multiple procedures, such as breast reconstruction, vaginal prolapse and ventral hernia repair [29]. Although there are differences in demographics, benefits, and cost of treatments between these procedures and stoma closure, the alignment of findings further reiterates that biological mesh prophylaxis is cost ineffective. However, current understanding on the risk and prevention of incisional hernias is limited [31]. In light of the benefit of prophylactic meshes in reducing rates of incisional hernias, this analysis provides the opportunity for the development of, this analysis lays the groundwork to better inform evidence-based algorithms for the role of prophylactic mesh in the future.

### 4.1. Limitations

The main limitation of this economic evaluation was the assumptions that had to be made regarding the costs of each complication. Notable absences of cost data were the unavailability of information on the costs of a seroma aspiration, and the lack of a formal method for calculating the extra cost of an operation when additional time is required. With regard to the ROCSS trial, complication data is limited to the 2-year follow up period. Further follow-up would have provided data on the longer-term effects and costs of increasing hernia complications, notable patient symptoms, and further surgery, and facilitate the measurement of the NHS resources that would be used. The natural progression of an incisional hernia is for symptoms to get worse over time. It is probable that a longer timeframe would give a more accurate indication of the effect of incisional hernias on quality of life, these differences may then be reflected in the average QoL scores of the cohorts.

As the ROCSS trial was a blinded RCT with a sample size of 790 patients, primarily conducted across 35 UK hospitals, the results of this economic evaluation are generalisable across the NHS. Findings indicate that biological mesh prophylaxis should not be routinely used during stoma closure and can be used to reliably inform NICE guidelines. However, these findings may not be generalised internationally, due to significant differences in healthcare quality and accessibility. With the majority of patients suffering from colorectal disease over the age of 50, the mean age (58.7) of patients included in this study ensures the results of this economic evaluation are generalisable for the typical patient undergoing stoma closure.

### 4.2. Contribution to the Literature

The clinical efficacy and cost-effectiveness of mesh prophylaxis for prevention of hernias remains a continuous debate in the literature. While several studies have shown promising results, the evidence base for the use of mesh for prophylaxis is weak. The UK based ROCSS randomised controlled trial is the first and only investigation providing high-quality evidence on the clinical efficacy of biological mesh prophylaxis for the prevention of hernias following stoma closure surgery.

Prior to this study, literature searches revealed that no economic evaluation had been performed for this intervention from the NHS, nor international perspective. Therefore, as the first economic evaluation of this intervention, this study is of significant contribution to current literature. Importantly, this cost-utility analysis acts as initial evidence to inform future NICE guidelines for the prevention of incisional hernias following stoma closure.

## 5. Conclusions

This CUA demonstrates that biological mesh prophylaxis is beneficial, yet more costly than standard suture closure alone, in the prevention of incisional hernias following stoma reversal surgery. Although not cost-effective, the benefit in reducing significant complications indicates the need for further risk stratification, to explore the cost-effectiveness of mesh prophylaxis for patients at greater risk of morbidity. Furthermore, given the supposed cost-effectiveness of synthetic mesh prophylaxis, more research is required to investigate the benefits and challenges with synthetic meshes, reduce intervention costs, and identify individualized use-case for the prevention of hernias using mesh prophylaxis.

## Figures and Tables

**Figure A1:**
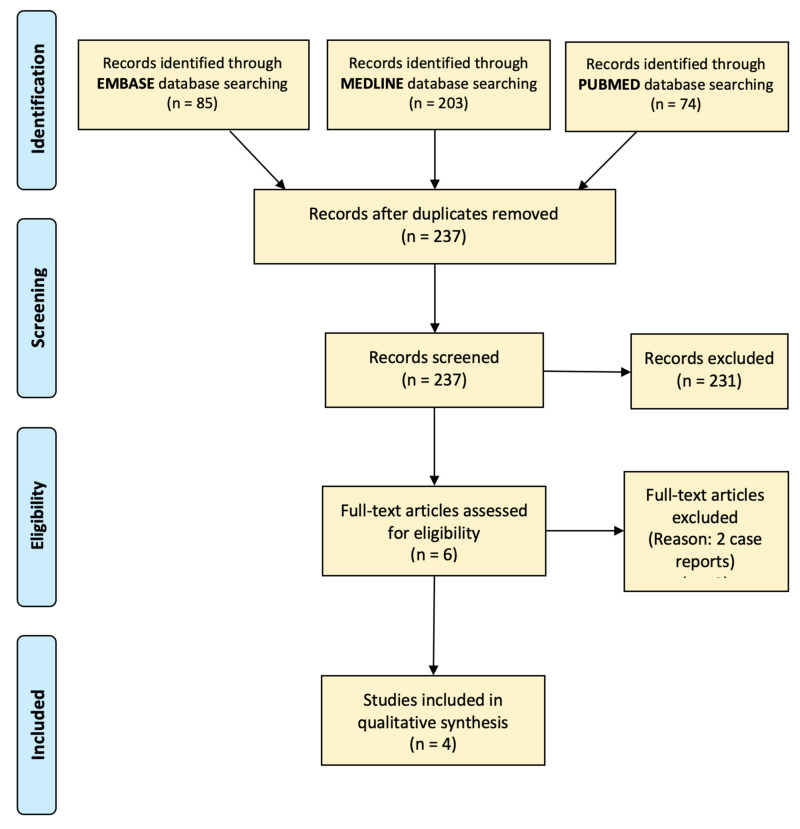
PRISMA flowchart for the systematic search strategy employed for the literature review. 2 databases were searched, yielding 4 relevant articles.

## Data Availability

All data used are references and are available within the manuscript or appendix.

## Appendix B

**Table A1:** Inclusion and exclusion criteria for the literature review.

Inclusion Criteria	Exclusion Criteria
Randomised controlled trials	Non-English studies
Retrospective cohort studies	Case reports
Cost analyses	Case series
	Conference Abstracts
	Studies comparing mesh prophylaxis and closure with sutures alone for procedures other than stoma closure surgery

## Appendix C

**Table A2:** Costs of healthcare resources required for stoma closure and associated complications.

Treatment Component	Unit Cost [GBP] (Year)	Explanation of Calculation	Discount Rate	Present Value (GBP)	Reference
Stoma Closure	£4247.60 (2021)	Weighted average of FF34B Distal Colon Procedures and FF22C Major Small Intestine Procedures based on the proportion of ileostomy and colostomy closure	-	£4247.60	[32]
Stoma Closure with Prophylactic Biological Mesh Reinforcement (Mesh)	£1650 (2017)	The average cost of biological mesh	1.035	£1893.41	[21]
Stoma Closure with Prophylactic Synthetic Mesh Reinforcement (Mesh)	£33 (2017)	The average cost of a synthetic mesh	1.035	£37.87	[21]
Stoma Closure with Prophylactic Biological Mesh Reinforcement (Extra time)	£20 (2019) £16 (2009)	The median extra duration of surgery (20 min) was multiplied by an average of the different estimated costs/min of operating theatres. Average cost/min = £22.80 Total cost = £456	1.035 1.035	£21.42 £24.18	[32] [33]
GP Consultation	£37.40 (2017)	Average cost of a 9 min GP consultation	1.035	£42.92	[34]
Co-amoxiclav	£1.69 (2021)	The standard treatment for stoma site wound infection. Modal drug tariff price. (Pack of 21, 1 tablet 3 times daily for 7 days)	-	£1.69	[23]
Wound dressings	£12 (2020)	Average cost of wound dressings	1.035	£12.42	[35]
Seroma drainage	£1419 (2021)	Most seromas need no treatment. Abscess drainage is used as a proxy for the costs of seroma drainage. Percutaneous Single Drainage of Abdominal Abscess	-	£1419	[35]
Incisional hernia repair	£3085 (2021)	Stoma site incisional hernias are by definition complex hernias. FF60C Complex Hernia Procedures with CC Score 1–2	-	£3085	[35]

## Appendix D

**Table A3:** Calculations conducted to obtain the total cost associated with each terminal node in the prophylactic biological mesh reinforcement arm of the decision tree *.

Complication	Cost Calculation	Total (GBP)
Wound infection + hernia (C1)	(0.2 * × [Colostomy Closure + additional time costs] + 0.8 × [Ileostomy Closure + additional time costs]) + Cost of biological mesh + Cost of GP Consultation + Cost of co-amoxiclav + Cost of wound dressings + 0.51 × Cost of incisional hernia repair	£8339.45
Wound infection + no hernia (C2)	(0.2 × [Colostomy Closure + additional time costs] + 0.8 × [Ileostomy Closure + additional time costs]) + Cost of biological mesh + Cost of GP Consultation + Cost of co-amoxiclav + Cost of wound dressings	£6654.04
Seroma + hernia (C3)	(0.2 × [Colostomy Closure + additional time costs] + 0.8 × [Ileostomy Closure + additional time costs]) + Cost of biological mesh + 0.067 × Cost of seroma drainage + 0.51 × Cost of incisional hernia repair	£8380.83
Seroma + no hernia (C4)	(0.2 × [Colostomy Closure + additional time costs] + 0.8 × [Ileostomy Closure + additional time costs]) + Cost of biological mesh + 0.067 × Cost of seroma drainage	£6695.41
No short-term complication + Hernia (C5)	(0.2 × [Colostomy Closure + additional time costs] + 0.8 × [Ileostomy Closure + additional time costs]) + Cost of biological mesh + 0.51 × Cost of incisional hernia repair	£8282.42
No complication (C6)	(0.2 × Colostomy Closure + additional time costs] + 0.8 × [Ileostomy Closure + additional time costs]) + Cost of biological mesh	£6957.01

***** 20% of the patients in the study were having a colostomy closure. 80% were having an ileostomy closure. 51% of hernias require treatment due to symptoms. Only 6.7% of seromas require treatment.

**Table A4:** Calculations conducted to obtain the total cost associated with each terminal node in the control arm of the decision tree.

Complication	Cost Calculation	Total (GBP)
Wound infection + hernia (F1)	(0.2 × Colostomy Closure + 0.8 × Ileostomy Closure) + Cost of GP Consultation + Cost of co-amoxiclav + Cost of wound dressings + 0.51 × Cost of incisional hernia repair	£5990.04
Wound infection + no hernia (F2)	(0.2 × Colostomy Closure + 0.8 × Ileostomy Closure) + Wound infection costs (GP Consultation + co-amoxiclav + wound dressings)	£4304.63
Seroma + hernia (F3)	(0.2 × Colostomy Closure + 0.8 × Ileostomy Closure) + 0.067 × Cost of seroma drainage + 0.51 × Cost of incisional hernia repair	£6031.41
Seroma + no hernia (F4)	(0.2 × Colostomy Closure + 0.8 × Ileostomy Closure) + 0.067 × Cost of seroma drainage	£4346.00
No short-term complication + Hernia (F5)	(0.2 × Colostomy Closure + 0.8 × Ileostomy Closure) + 0.51 × Cost of incisional hernia repair	£5933.01
No complication (F6)	(0.2 × Colostomy Closure + 0.8 × Ileostomy Closure)	£4247.60

## Appendix E

**Table A5:** Calculations conducted to obtain the expected QALY at each decision node in the tree.

Treatment Option	QALY Calculation	Total QALY
Prophylactic Biological Mesh Reinforcement	$0.79\times \frac{1}{12}+0.86\times \frac{11}{12}+0.85\times \frac{12}{12}$	1.70416
Standard Closure	$0.81\times \frac{1}{12}+0.84\times \frac{11}{12}+0.85\times \frac{12}{12}$	1.6875

The number of patients with wound infections is measured at 30 days postoperatively; the number of patients with a formed seroma is measured at 1 year post randomisation; and the number of patients with a clinically detectable hernia is measured at 2 years post-randomisation. Quality of life scores were assessed at these same time points. We have taken the quality-of-life scores for the time periods to reflect the utility for the different complications measured at these time points.

## Appendix F

**Table A6:** Reasons for exclusion of certain secondary outcomes.

Secondary Outcome	Reason for Exclusion as Specific Node	Group into Other Outcomes
Wound infection at 12 months	Most wound infections show up within the first 30 days after surgery. The outcome table in the study reflects this.	No
Radiological hernia at 12 months	Demographic details for patients included in the analysis for the radiological hernia outcome showed no clinically important differences	No
Symptomatic hernia at 12 months	Hernia already measured in primary outcome	No
Symptomatic hernia at 24 months	Hernia already measured in primary outcome	No
Surgical re-intervention at stoma site	Reason for re-intervention is not specified	No

## Appendix G

**Table A7:** Calculations for the incidence of hernia given wound infection.

Treatment Option	Incidence of Hernia in Study	Incidence of Wound Infection	Relative Risk of Hernia Given Wound Infection (Source)	Calculation
Prophylactic mesh	$\frac{39}{323}$	$\frac{60}{371}$	3.68 [36]	$\frac{39}{323}=\frac{60}{371}\times \frac{3.68x}{323}+\frac{311}{371}\times \frac{x}{323}$ Solving for x x=0.084
Standard closure	$\frac{64}{327}$	$\frac{49}{369}$	3.68 [36]	$\frac{64}{327}=\frac{49}{369}\times \frac{3.68x}{327}+\frac{320}{369}\times \frac{x}{327}$ Solving for x x=0.144

## Appendix H

**Table A8:** Calculations for the utility of having a hernia based on expected difference in QoL score from literature.

Incidence of Hernia	Hernia Effect on QoL Score(Source)	Calculation
$\frac{39}{323}$	25% decrease [37]	$0.85=\frac{39}{323}\times \frac{0.75x}{100}+\frac{284}{323}\times \frac{x}{100}$ Solving for x x=0.87

## Appendix I

**Table A9:** NMB * calculations for the original model and the two sensitivity analyses.

	∆	ICER (GBP/QALY)	NMB (GBP)	Calculation
Original model	∆C = £2139.27 ∆E = 0.016	£128,356.25	−£1639.27	= (30,000 × 0.016) − 2139.27
Sensitivity analysis with adjusted QALYs	∆C = £2139.27 ∆E = 0.0317	£67,559.33	−£1189.32	= (30,000 × 0.0317) − 2139.27
Sensitivity analysis with all incisional hernias treated	∆C = £2019.39 ∆E = 0.016	£121,163.37	−£1519.39	= (30,000 × 0.016) − 2019.39
Sensitivity analysis using synthetic mesh prophylaxis	∆C = £335.68 ∆E = 0.016	£20,140.88	+£144.32	= (30,000 × 0.016) − 335.68

* NMB = Rc × ΔE − ΔC.

**Table A10:** NHB * calculations for the original model and the two sensitivity analyses.

	∆	ICER (GBP/QALY)	NHB (GBP)	Calculation
Original model	∆C = £2139.27 ∆E = 0.016	£128,356.25	−0.055	$=0.016-\;\frac{2139.27}{30000}$
Sensitivity analysis with adjusted QALYs	∆C = £2139.27 ∆E = 0.0317	£67,559.33	−0.040	$=0.0317-\frac{2139.27}{30000}$
Sensitivity analysis with all incisional hernias treated	∆C = £2019.39 ∆E = 0.016	£121,163.37	−0.051	$=0.016-\;\frac{2139.27}{30000}$
Sensitivity analysis with synthetic mesh	∆C = £335.68 ∆E = 0.016	£20,140.88	+0.0048	$=0.016-\;\frac{335.68}{30000}$
